# Specific Features of the Coagulopathy Signature in Severe COVID-19 Pneumonia

**DOI:** 10.3389/fmed.2021.675191

**Published:** 2021-08-04

**Authors:** Mathieu Blot, Emmanuel de Maistre, Abderrahmane Bourredjem, Jean-Pierre Quenot, Maxime Nguyen, Belaid Bouhemad, Pierre-Emmanuel Charles, Christine Binquet, Lionel Piroth

**Affiliations:** ^1^Infectious Diseases Department, Dijon Bourgogne University Hospital, Dijon, France; ^2^Lipness team, INSERM Research Center LNC-UMR1231 and LabEx LipSTIC, University of Burgundy, Dijon, France; ^3^Laboratory of Hemostasis, Dijon Bourgogne University Hospital, Dijon, France; ^4^INSERM, CIC1432, Clinical Epidemiology unit, Dijon, France; ^5^Dijon Bourgogne University Hospital, Clinical Investigation Center, Clinical Epidemiology/Clinical trials unit, Dijon, France; ^6^Department of Intensive Care, Dijon Bourgogne University Hospital, Dijon, France; ^7^Anesthesiology and Critical Care Department, Dijon Bourgogne University Hospital, Dijon, France

**Keywords:** COVID-19, pneumonia, acute respiratory distress syndrome, coagulopathy, thrombosis, VCAM1

## Abstract

**Rationale:** COVID-19 displays distinct characteristics that suggest a unique pathogenesis. The objective of this study was to compare biomarkers of coagulopathy and outcomes in COVID-19 and non-COVID-19 patients with severe pneumonia.

**Methods:** Thirty-six non-COVID-19 and 27 COVID-19 non-immunocompromised patients with severe pneumonia were prospectively enrolled, most requiring intensive care. Clinical and biological characteristics (including plasma biomarkers of coagulopathy) were compared.

**Results:** At similar baseline severity, COVID-19 patients required mechanical ventilation (MV) for significantly longer than non-COVID-19 patients (*p* = 0.0049) and more frequently developed venous thrombotic complications (*p* = 0.031). COVID-19 patients had significantly higher plasma concentrations of soluble VCAM1 (sVCAM1) (5,739 ± 3,293 vs. 3,700 ± 2,124 ng/ml; *p* = 0.009), but lower levels of D-dimers, vWF-A2, sICAM1, sTREM1, VEGF, and P-selectin, compared to non-COVID-19 patients. Principal component analysis identified two main patterns, with a clear distinction between non-COVID-19 and COVID-19 patients. Multivariable regression analysis confirmed that sVCAM1 rising levels were independently associated with a longer duration of MV. Finally, we identified close correlations between sVCAM1 and some features of COVID-19 immune dysregulation (ie. CXCL10, GM-CSF, and IL-10).

**Conclusion:** We identified specific features of the coagulopathy signature in severe COVID-19 patients, with higher plasma sVCAM1 levels, that were independently associated with the longer duration of mechanical ventilation.

**Clinical Trial Registration:**ClinicalTrials.gov, identifier: NCT03505281.

## Introduction

Severe acute respiratory syndrome coronavirus 2 (SARS-CoV-2) is responsible for severe pneumonia and acute respiratory distress syndrome (ARDS) in its most severe form. Thrombotic complications have frequently been reported in coronavirus disease 2019 (COVID-19) patients. Coagulopathy was quickly identified as part of the pathogenesis of COVID-19 (1). Elevated levels of D-dimer and fibrin (ogen) degradation products are associated with outcomes in COVID-19 and suggest major coagulation activation (1–4). In addition, endothelial dysfunction and platelet activation are two further hallmarks of the most severe forms of COVID-19 (4–6). On the other hand, coagulopathy is also a classical complication of bacterial pneumonia and ARDS, where D-dimer elevation and platelet decrease are common features of the most severe forms (7, 8). However, COVID-19 displays peculiar characteristics that suggest a unique pathogenesis (9, 10). First, COVID-19-related ARDS is characterized by its protracted course, lasting twice as long as ARDS of other origins (11, 12). Second, our team and others recently identified a unique cytokine response with higher concentrations of CXCL10 and GM-CSF in COVID-19 patients that were independently associated with outcomes (13–16). In addition, COVID-19 ARDS patients experience significantly more thrombotic complications than non-COVID-19 ARDS patients, mainly pulmonary embolism and deep vein thrombosis (2). Autopsy findings have shown thrombosis and microangiopathy in the small vessels and capillaries of the lungs (17, 18) that could contribute to this poor outcome. However, comparisons between COVID-19 and pneumonia of other origins are scarce (2).

Thus, our study addressed this issue by comparing coagulopathy-related biomarkers between non-COVID-19 and COVID-19 patients with severe pneumonia and studying the association between such biomarkers and clinical outcomes.

## Materials and Methods

### Study Design and Patient Selection

The present work is a prospective, exploratory ancillary study of the ongoing LYMPHONIE project (registered with ClinicalTrials.gov under the number NCT03505281), initiated in November 2018 at the University Hospital of Dijon-Bourgogne (France). Patients were eligible if they had severe community-acquired pneumonia (CAP) associating: (1) pneumonia (≥2 acute signs including cough, purulent sputum, dyspnea, chest pain, temperature <35 or ≥38.5°C, and new radiological pulmonary infiltrate); and (2) severity defined by at least two criteria of the quick-Sequential Organ Failure Assessment (SOFA) score (systolic blood pressure ≤ 100 mm Hg, respiratory rate ≥22, Glasgow score <15) and/or the need for mechanical ventilation (MV) and/or vasopressors; and (3) diagnosed within 48 hours following admission. Non-inclusion criteria were: <18 years, pregnant women, persons under legal protection, decision to limit care, known immune deficiency, chronic disorder known to cause deep lymphopenia (i.e. cirrhosis, lympho- or myeloproliferative syndrome, solid cancer or active systemic lupus), hospitalization for sepsis within the previous 3 months. Non-COVID-19 CAP patients were included until February 20, 2020, one month before the COVID-19 pandemic started in Burgundy, France. COVID-19 patients were eligible if they tested positive for SARS-CoV-2 by reverse transcriptase-polymerase chain reaction (RT-PCR) on one respiratory sample. Oral consent was obtained from the patient or their legal representative. Approval was obtained from the ethics committee (Comité de Protection des Personnes SUD MEDITERRANEE V; 2017-A03404-49). Ethylenediamine tetraacetic acid blood samples were obtained after inclusion of the patient and within 48 hours of hospital admission. After centrifugation of blood samples at 2,000 × g for 10 min at 4°C, plasma was collected and stored at −80°C in the biological resource center (CRB Ferdinand Cabanne; NF S96-900 certification).

### Objectives of the Study

The main objective of this study was to compare plasma biomarkers of coagulopathy between non-COVID-19 and COVID-19 with severe pneumonia.

The secondary objective was to study the association between the biomarkers of coagulopathy, and clinical outcomes potentially related to coagulopathy.

### Variables of Interest, Clinical Outcomes, and Data Collection

Clinical and biological parameters, Charlson comorbidity index and severity scores (SOFA (19), Simplified Acute Physiology Score (SAPS II) (20) and Pneumonia Severity Index (PSI) (21)) were calculated at the time of inclusion. ARDS was defined according to the Berlin definition (22), and septic shock was defined as persistent hypotension requiring vasopressors and a serum lactate level >2 mmol/L despite adequate volume resuscitation. Clinical outcomes were recorded up to 30 days after admission, namely: deep vein thrombosis, pulmonary embolism, 30-day mortality, hospital- and ICU- length of stay and duration of MV. Venous thrombotic events were diagnosed as part of routine care, either with computed tomographic pulmonary angiography (pulmonary embolism) or vascular ultrasonography (deep vein thrombosis). Dedicated clinical research assistants collected all data using a standardized electronic case report form. Automatic checks were generated for missing or incoherent data.

### Plasma Biomarkers of Coagulopathy Measurement

Thirteen analytes were quantified in plasma using a Luminex® Human Magnetic assay (R&D Systems, USA) according to the manufacturer's instructions: endothelial and procoagulant response [D-dimer, von Willebrand factor A2 (vWF-A2), u-plasminogen activator (uPA), tissue factor pathway inhibitor (TFPI), soluble thrombomodulin (sTM) receptor from endothelial cells participating in the control of thrombin, soluble triggering receptor expressed on myeloid cells 1 (sTREM-1), soluble intercellular adhesion molecule 1 (sICAM-1) and soluble vascular cell adhesion molecule 1 (sVCAM-1) from endothelial cells, vascular endothelial growth factor (VEGF)], and platelet activation [C-X-C motif chemokine ligand (CXCL) 4, soluble P-selectin, platelet derived growth factor (PDGF)-AA, PDGF-AB/BB]. In addition IL-1β, IL-6, IL-10, CXCL-10, GM-CSF and TNF-α, were also quantified in plasma using a Luminex® Human Magnetic assay (R&D Systems, USA) to study the potential link between immune response and coagulopathy (16). Biomarkers measurements were performed on the same day, by the same person.

### Statistical Analysis

Data are described according to COVID-19 status (i.e. non-COVID-19 vs. COVID-19 patients). Continuous variables are expressed as mean ± standard deviation (SD) or median and inter-quartile range (IQR), according to their distribution, and categorical variables as number and percentage. Univariate comparisons were performed using Student's test for means, Wilcoxon Mann-Whitney test for medians and IQRs, and the Chi-square test (or Fisher's exact test when appropriate) for percentages. Coagulopathy biomarkers are presented as boxplots to visualize potential associations with COVID-19 status. The Benjamini–Hochberg procedure was used to control the false discovery rate (FDR) induced by multiple comparisons (23). For each test, a Q-value (adjusted *p*-value) was calculated, representing the minimum FDR at which the statistical test may be considered as a true positive (i.e. statistically significant given the multiplicity of tests). A result was deemed false positive when the corresponding Q-value was >0.05. To account for potential confounders, multivariable linear regressions considering the relevant coagulopathy biomarkers as an independent variables and COVID-19 status as the main explicative covariate were performed. These models were systematically adjusted for the most clinical relevant variables (i.e. age, sex, Charlson comorbidity index and SOFA score). Absence of serial correlation and heteroscedasticity were assessed using the DW statistic (24) and the White test (25), respectively.

Principal component analysis (PCA) was used to identify potentially significant patterns of 30 variables: the 13 coagulopathy biomarkers and the following 17 clinical and biological variables: PSI, SOFA, CRP, PCT, Creatinine, NT-ProBNP, leucocytes, neutrophils, lymphocytes, monocytes, platelets, prothrombin time (PT), PaO2:FiO2, lactate, MV duration, ICU and hospital length of stay. PCA identifies factors, called principal components, that induce the most variation in the overall data (26). These factors can be expressed as a linear combination of the correlated original variables (OVs). By inversing these formulas, we can express each OV as a linear combination of the factors and coefficients defining these linear combinations may be interpreted as correlation coefficients. Moreover, each factor describes a percent of variation in the OVs. The number of factors to retain was determined using the scree plot (27) and the clinical interpretability of factors (28). Besides, patient OV data can be projected on the planes defined by the factors retained, making it possible to observe patient patterns in a two-dimensional plot.

To strengthen interpretation, Spearman correlations were computed between coagulopathy biomarkers and the most pertinent patient characteristics associated with COVID-19 status in univariate analyses and PCA patterns. Then, multivariable linear regressions were applied to estimate the associations between the selected biomarkers of coagulopathy and MV duration, considered as the independent variable. These models were systematically adjusted for the most pertinent variables (i.e. age, pre-hospital anticoagulation therapy, COVID-19 status, and SOFA score). As above, absence of serial correlation and heteroscedasticity were assessed using the DW statistic (24) and the White test (25), respectively. The interaction between COVID-19 status and the selected biomarkers of coagulopathy was systematically tested. The proportion of variance explained by the models was quantified using the R^2^ coefficient. Measures of association are expressed as mean differences ± standard error (SE). Finally, a heatmap correlation matrix were built between biomarkers of coagulopathy and plasma cytokines concentrations. A *p*-value < 0.05 was considered statistically significant. Analyses were performed using SAS version 9.4 (SAS Institute Inc., Cary, NC, USA).

## Results

### Characteristics of the Study Population

Sixty-three patients were enrolled (36 in the non-COVID-19 group, and 27 in the COVID-19 group). Bacterial, viral or mixed etiologies were proven in 10 (28%), 10 (28%) and 3 (8%) patients from the non-COVID-19 group, respectively (Supplementary Table 1). Mean age was marginally lower in the COVID-19 group as compared to the non-COVID-19 group (62.5 ± 10.9 vs. 68.0 ± 13.0; *p* = 0.07). Other demographic and comorbidity data were not statistically different between the two groups, with the exception of the prehospital anticoagulation therapy, which was more frequent in non-COVID-19 patients (*p* = 0.033; Table 1). Although the PaO_2_:FiO_2_ ratio and SOFA score were not different between groups (*p* = 0.35 and *p* = 0.52, respectively), COVID-19 patients less often had septic shock (0 vs. 11 (31%); *p* = 0.0015). In COVID-19 patients, we observed significantly lower arterial lactates (*p* = 0.01), serum creatinine (*p* = 0.02), NT-proBNP (*p* = 0.0003), procalcitonin (*p* = 0.007) and CRP (*p* = 0.004) levels (Table 1). White blood cell and platelet counts did not differ significantly between groups, except for monocytes, which were lower (*p* = 0.05) in COVID-19 patients (Table 1). All patients were treated with antibiotics.

**Table 1:** Baseline characteristics and outcomes of the study population (LYMPHONIE study, 2018–2020).

		**Study group**		
	**Normal range**	**non-COVID-19**	**COVID-19**	***P***
		***N* = 36**	***N* = 27**	
**Demographics**				
Age (years), mean ± SD		68.0 ± 13.0	62.5 ± 10.9	0.07
Male sex, *n* (%)		29 (81%)	17 (63%)	0.12
Body-mass index (kg/m^2^), mean ± SD		29.1 ± 7.1	30.7 ± 8.1	0.43
**Chronic comorbidities**				
Cardiovascular disease, *n* (%)		12 (33%)	5 (19%)	0.25
Pulmonary disease, *n* (%)		12 (33%)	5 (19%)	0.25
Chronic renal disease, *n* (%)		2 (6%)	1 (4%)	0.73
Cerebrovascular disease, *n* (%)		5 (14%)	3 (11%)	0.74
Diabetes mellitus, *n* (%)		10 (28%)	2 (7%)	0.28
Charlson score, mean ± SD		1.5 ± 2.0	0.9 ± 0.9	0.12
Prehospital anticoagulation therapy, *n* (%)		6 (17%)	0	0.033
Prehospital antiplatelet therapy, *n* (%)		9 (25%)	6 (22%)	1
**Severity at hospital admission**				
Septic shock, *n* (%)		11 (31%)	0	0.0015
ARDS, *n* (%)		23 (64%)	25 (93%)	0.015
Pneumonia Severity Index, mean ± SD		117.8 ± 38.6	94.2 ± 27.1	0.006
SAPS II, mean ± SD		23.8 ± 9.9	19.4 ± 9.4	0.08
SOFA score, mean ± SD		7.2 ± 3.6	6.7 ± 2.0	0.52
**Biological findings on admission**				
ASAT (IU/l), mean ± SD	15–37	86.3 ± 92.4	86.2 ± 54.6	0.99
Serum Creatinine (μmol/l), mean ± SD	59–104	132.9 ± 93.3	90.2 ± 40.7	0.02
NT-ProBNP (pg/ml), mean ± SD	<125	5687 ± 7694	2225 ± 6257	0.05
PaO_2_:FiO_2_ (mm Hg), mean ± SD	≥400	123.7 ± 54.9	136.2 ± 49.8	0.35
Arterial pH (mm Hg), mean ± SD	7.35–7.45	7.35 ± 0.11	7.40 ± 0.07	0.07
Serum Bicarbonate (mmol/l), mean ± SD	20–29	24.0 ± 5.1	24.6 ± 3.1	0.59
Lactate level (mmol/l), mean ± SD	0.5-2.0	2.6 ± 1.9	1.7 ± 0.7	0.01
C-reactive protein (mg/l), mean ± SD	<3.2	259.9 ± 156.8	172.8 ± 62.9	0.004
Procalcitonin (μg/L), mean ± SD	<0.10	32.4 ± 61.9	2.6 ± 6.6	0.007
PT (%), mean ± SD	**>**70%	68 ± 20	91 ± 9	<0.001
aPTT, mean ± SD	0.7–1.2	1.31 ± 0.34	1.14 ± 0.18	0.18
Platelets (x10^9^/l), mean ± SD	150–400	210 ± 873	241 ± 929	0.18
Leukocytes (x10^6^/l), mean ± SD	3.8–9.5	12.2 ± 6.4	10.8 ± 5.7	0.38
Neutrophils (x10^6^/l), mean ± SD	1.7–5.8	11.3 ± 5.5	9.4 ± 5.6	0.18
Lymphocytes (x10^6^/l), mean ± SD	1.07–4.03	0.64 ± 0.40	0.78 ± 0.38	0.16
Monocytes (x10^6^/l), mean ± SD	0.2–0.7	0.61 ± 0.46	0.44 ± 0.25	0.05
**Treatments**				
Antibiotic multitherapy, *n* (%)		29 (81%)	17 (63%)	0.12
Corticosteroids, *n* (%)		16 (44%)	16 (59%)	0.24
Hydroxychloroquine, *n* (%)		0	10 (37%)	
Remdesivir, *n* (%)		0	3 (11%)	
Invasive mechanical ventilation, *n* (%)		24 (67%)	23 (85%)	0.09
ECMO, *n* (%)		0	1 (4%)	0.43
Renal replacement therapy, *n* (%)		5 (14%)	0	0.065
Vasopressors, *n* (%)		19 (53%)	19 (70%)	0.16
**Outcomes at 30 days**				
Venous thrombotic complications		2 (6%)	7 (26%)	0.0312
Deep vein thrombosis, *n* (%)		1 (3%)	5 (19%)	0.07
Pulmonary embolism, *n* (%)		1 (3%)	3 (11%)	0.30
ICU admission, n (%)		32 (89%)	274 (100%)	0.12
Median ICU length of stay (days) (IQR)		13 (4-20)	20 (12-29)	0.0274
Median days of mechanical ventilation (IQR)		4 (0-15)	15 (7-22)	0.0049
Median hospital length of stay (days) (IQR)		21 (13-30)	29 (20-30)	0.087
30 day-mortality, *n* (%)		2 (6%)	1 (4%)	1

*ARDS, acute respiratory distress syndrome; SAPS II, Simplified Acute Physiology Score II; SOFA, Sequential Organ Failure Assessment; ASAT, aspartate aminotransferase; NT-proBNP, N-Terminal Fragment of the prohormone Brain-Type Natriuretic Peptide; PaO_2_:FiO_2_, arterial pressure of oxygen / oxygen inspiratory fraction; NK, Natural killer; ECMO, extracorporeal membrane oxygenation; ICU, intensive care unit*.

### At Similar Baseline Respiratory Severity, COVID-19 Patients Were More Likely to Have Venous Thrombotic Complications and Had a Significantly Longer Duration of MV

Venous thrombotic events (i.e. deep vein thrombosis or pulmonary embolism) were more frequently diagnosed in COVID-19 patients than in non-COVID-19 patients [7 (26%) vs. 2 (6%); *p* = 0.0312)], with a median time from admission to diagnosis of 8 (IQR = 6–11) and 11 (IQR = 9–14) days respectively (*p* = 0.86). COVID-19 patients had a significantly longer duration of MV (15 days, IQR = 7–22 vs. 4 days, IQR = 0–14.5; *p* = 0.0049), as well as longer ICU stay (*p* = 0.0274) (Table 1). The 30-day mortality rate was 6% (*n* = 2) in the non-COVID-19 group and 4% (*n* = 1) in the COVID-19 group (*p* = 1.00).

### COVID-19 Patients Displayed a Unique Plasma Signature of Coagulopathy-Associated Biomarkers

**Figure 1:**
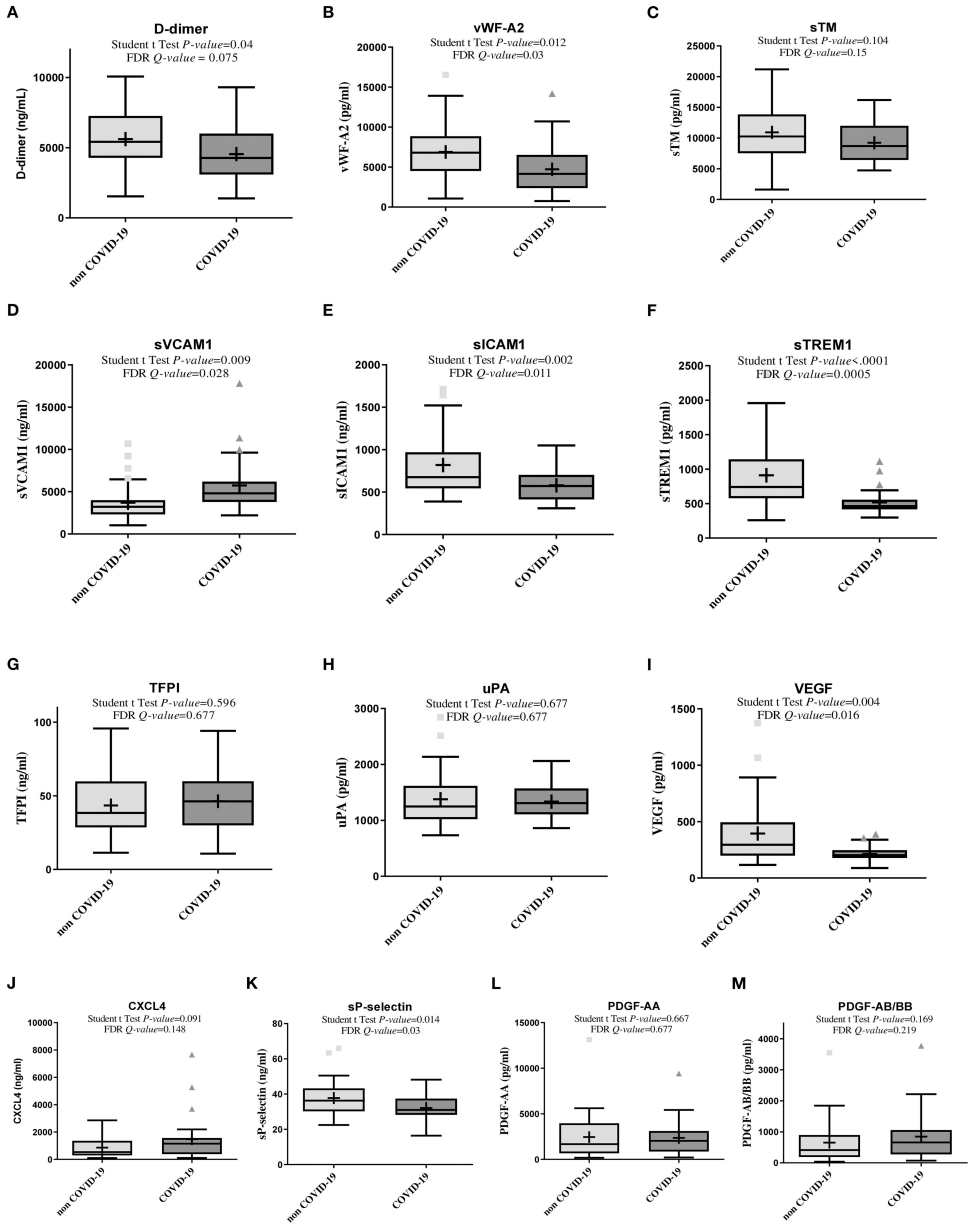
Box plot showing plasma concentrations of biomarkers of coagulopathy in non-COVID-19 and COVID-19 patients (Lymphonie study 2018–2020). Plasma concentration of biomarkers of coagulation and/or endothelium and platelet activation was measured within 48 hours of hospitalization in 36 non-COVID-19 and 27 COVID-19 patients with severe pneumonia. COVID-19 patients had significantly higher plasma concentrations of sVCAM1 **(D)**, lower plasma concentration of D-dimers, vWF-A2, sICAM1, sTREM1, VEGF, sP-selectin **(A,B,E,F,I,K)** and a non-significant difference for plasma levels of sTM, TFPI, uPA, CXCL4, PDGF-AA and PDGF-AB/BB. For each cytokine, *P*-values from Student *t* test and *Q*-value of the false discovery rate (FDR) are indicated. COVID-19, coronavirus disease 2019; CXCL4, platelet C-X-C motif chemokine ligand 4; FDR, false discovery rate; PDGF, platelet derived growth factor; sICAM-1, soluble intercellular adhesion molecule 1; sTREM-1, soluble triggering receptor expressed on myeloid cells 1; sVCAM-1, soluble vascular cell adhesion molecule 1; sTM, soluble thrombomodulin; TFPI, tissue factor pathway inhibitor; uPA, u-plasminogen activator; VEGF, vascular endothelial growth factor; vWF-A2, von Willebrand factor A2 (LYMPHONIE study, 2018–2020).

COVID-19 patients had higher plasma sVCAM1 (Figure 1). However, in COVID-19 patients, we observed lower plasma levels of D-dimers, vWF-A2, sICAM1, sTREM1, VEGF, and sP-selectin as compared to non-COVID-19 patients, and a non-significant difference for plasma levels of sTM, TFPI, uPA, CXCL4, PDGF-AA and PDGF-AB/BB (Figure 1, Supplementary Table 2).

### PCA identified Two Patterns Linking Coagulopathy-Associated Biomarkers and Outcomes, With a Clear Distinction Between Non-COVID-19 and COVID-19 Patients

**Figure 2:**
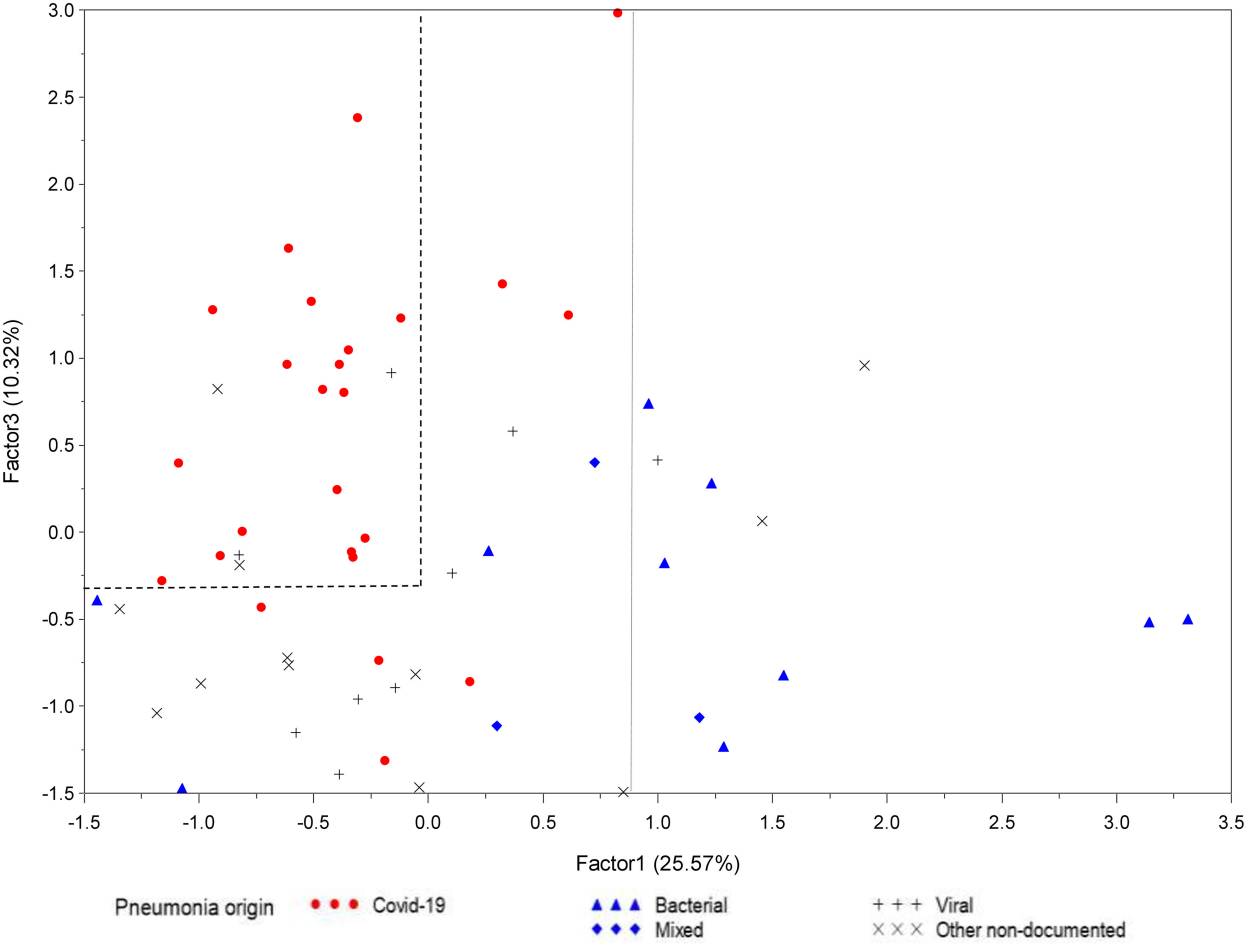
Two-dimensional score plot of principal component analysis according to pneumonia etiology (Lymphonie study 2018–2020). Principal component analysis (PCA) was used to identify potentially significant patterns of 30 variables: 13 plasma biomarkers of coagulopathy and 17 clinical and biological variables from 63 patients with severe pneumonia [non-COVID-19 (*n* = 36), COVID-19 (*n* = 27)]. Factors 1 and 3 were used to build a two-dimensional score plot of PCA. There is a clear separation between COVID-19 patients (red circles) and non-COVID-19 patients: bacterial (blue triangles), mixed (blue diamonds), viral (gray crosses) and other non-documented severe community acquired pneumonia (gray Xs). Bacterial and mixed severe pneumonia were represented by a high factor 1, while COVID-19 patients had a high factor 3 (LYMPHONIE study, 2018-2020).

In PCA, three factors were retained, which together accounted for 51.6% of all the information contained in the 30 correlated original variables (Supplementary Table 3, Supplementary Figure 1). Two of the three factors in the final pattern were associated with outcomes. Factor 1 associated (1) baseline severity and extra-respiratory organ dysfunction (SOFA and PSI scores, lactate, creatinine and NT-ProBNP levels) and (2) “classical” markers of coagulation and endothelium activation (i.e. D-dimer, vWF-A2, sTM, sICAM1, TREM1, TFPI, uPA, VEGF). Factor 3 associated: (1) outcomes (duration of MV, ICU and hospital length of stay) and (2) sVCAM1 (Supplementary Table 3). The projection of patient data onto the directions defined by Factors 1 and 3 showed a clear separation between COVID-19 and non-COVID-19 patients. By plotting patients according to different origins of pneumonia, we observed that most of the non-COVID-19 patients with proven bacterial (or mixed) origin had high Factor 1 values, while COVID-19 patients had high Factor 3 and low Factor 1 values (Figure 2). Factor 2 associated: (1) white blood cell and platelets levels and (2) markers of platelet activation and growth factors (ie. CXCL4, sP-selectin, PDGF-AA, PDGF-AB/BB) (Table 2). Planes defined by factor 2 did not enable any discrimination between patients according to COVID-19 status.

**Table 2:** Multivariable linear regression factors associated with duration of mechanical ventilation (variation in minutes) in the first 30 days of hospitalization among 63 patients with severe pneumonia (R^2^ = 41.3%) (LYMPHONIE study, 2018–2020).

**Variable**	**Mean diff**	**±SE**	***P***
COVID-19 (Yes/No)	8,186	3,463	0.022
sVCAM1 (per 3,000 ng/ml increase)	4,050	1,786	0.027
Age (per additional year)	−10.2	126.7	0.94
SOFA score (for each additional point)	1,978	513.0	0.0003
Pre-hospital anticoagulation therapy (Yes/No)	4,210	5,170	0.42

*For sVCAM1, an increase of 3,000 ng/ was considered, based on the standard deviation of sVCAM1 plasma levels in COVID-19 patients (i.e. 3,293 ng/ml). COVID-19, coronavirus disease 2019; SOFA, Sequential Organ Failure Assessment; SE, standard error; sVCAM1, soluble vascular cell adhesion molecule 1*.

### Plasma sVCAM1 Was Independently Associated With the Duration of Mechanical Ventilation

As the particular severity of COVID-19 patients was represented by a significantly longer duration of MV, we investigated whether coagulopathy-associated biomarkers was associated with this poor outcome. First, we observed a significant correlation between the duration of MV and sVCAM1 (*p* < 0.0001) and uPA (*p* = 0.001) (Supplementary Table 4). In multivariable linear regression, sVCAM1 was independently associated with a longer duration of MV (*p* = 0.027), after adjustment for age, COVID-19 status, SOFA score and prehospital anticoagulation therapy (Table 2). We estimated an excess of 2.8 ± 1.2 days of MV per increase of 3,000 ng/mL of sVCAM1 (*p* = 0.027).

In addition, multivariable analysis showed that COVID-19 status was independently associated with sVCAM1. In severe COVID-19 patients, we estimated a proper excess of 2,247 ± 695 ng/ml for sVCAM1 (*p* = 0.002), independently of age, sex, Charlson and SOFA scores (Supplementary Table 5). Autocorrelation and heteroscedasticity were non-significant in the models.

### sVCAM1 Was Strongly Correlated With CXCL10, GMCSF and IL-10 Concentrations

Since sVCAM1 is upregulated on immune-mediated activation, we investigated whether sVCAM1 was correlated with the production of cytokines. We observed strong correlations between sVCAM1 and each of CXCL10, GM-CSF and IL-10 (respectively r = 0.71, *p* < 0.0001; r = 0.66, *p* < 0.0001 and r = 0.70, *p* < 0.0001), but not with IL-1β, IL-6 and TNF-α (**Supplementary** Figure 2).

## Discussion

Our study comparing non-COVID-19 with COVID-19 patients with severe pneumonia identified two main results: (1) COVID-19 patients displayed a distinct coagulopathy signature, with significantly higher concentrations of sVCAM1, but lower concentrations of some classical biomarkers of coagulation, endothelial or platelet activation; (2) sVCAM1 was independently associated with the duration of MV and strongly correlated with some recently reported features of the dysregulated immune response in severe COVID-19 patients.

Patients with severe COVID-19 display particular characteristics as compared to pneumonia of other origins, notably with a higher risk of thrombotic complications in COVID-19 (2), and a longer duration of MV, as observed in our study. There is an urgent need to understand the peculiar pathogenesis of severe COVID-19, with a view to finding new therapeutic strategies likely to improve outcomes in the most severe forms (e.g. ARDS). Ackermann et al. showed that patients who died from COVID-19 had a distinctive vascular histologic pattern with severe endothelial injury associated with the presence of intracellular SARS-CoV-2 and peripheral T-cell infiltration (18). Different studies have highlighted complement activation (e.g. the C5aR1 axis (29)), platelet activation (4), immune-mediated thrombotic mechanisms (6), endothelial dysfunction, antiphospholipid antibody, and renin-angiotensin system dysregulation as important actors of COVID-19-associated coagulopathy (30). However, the pathophysiological process driving the poorer outcomes in severe COVID-19 pneumonia remains unclear, and comparisons with pneumonia of other origins are scarce.

In our study, we showed firstly that, regardless of baseline clinical severity, plasma concentrations of numerous biomarkers characterizing coagulation/endothelial activation (i.e. D-dimers, vWF-A2, sICAM1, sTREM1, VEGF) or platelet activation (sP-selectin) were elevated in both groups, but lower in COVID-19 compared to non-COVID-19 patients. Other markers (e.g. sTM, TFPI, uPA, CXCL4, PDGF-AA and PDGF-AB/BB) did not appear to differ between the two groups. Coagulopathy is a hallmark of ARDS regardless of its origin (8), and could contribute to the pathogenesis of lung injury and subsequent respiratory failure (31). Indeed, we observed that most of the biomarkers characterizing coagulation/endothelial and platelet activation were correlated with the baseline severity, in particular in proven bacterial pneumonia (higher Factor 1 in the PCA analysis), but not with the outcomes. In addition, the higher concentrations of D-dimers in non-COVID-19 patients could be explained by a greater activation of fibrinolysis, related to the higher rate of septic shock, as compared to COVID-19 patients. In contrast, even if these later frequently required vasopressors, they never met the criteria for septic shock in our study, considering the low concentrations of lactate.

However, plasma sVCAM1 was the only biomarker found to be higher in COVID-19 patients. Correlation and PCA analyses identified a distinct pattern linking sVCAM1 to the duration of MV, which represents the characteristic outcome associated with COVID-19 and a major concern for clinicians and health systems. Finally, multivariable analysis confirmed that sVCAM1 was independently associated with the duration of MV. Based on these analyses, we hypothesized that sVCAM1 could drive the dysregulated coagulopathy leading in turn to severe lung injury and prolonged need for MV in COVID-19 ARDS patients.

VCAM-1 is an integrin expressed in endothelial cells, but also on leukocytes, and is upregulated on immune-mediated activation (pro-inflammatory cytokines, including TNF-α and IFNs) (32). VCAM1 interacts with α4β1 integrins on leukocytes, and in turn, mediates their adhesion and activation to vascular endothelium and engages their migration (33). VCAM1 has been shown to regulate leukocyte trafficking and immune response against viral infections (34). Our results are consistent with vascular endothelial dysfunctions described in COVID-19 patients, and also mediated by SARS-CoV-2 adhesion on endothelial cells (35). However, in parallel, ICAM-1, a transmembrane integrin, mediates the migration of leukocytes into the inflamed tissues (36). Both integrins were found to be elevated in the serum of COVID-19 patients and related to severity of disease (37). Since circulating levels of these isoforms reflect enhanced expression of the respective adhesion molecules (38), we postulate that overexpression of VCAM1 in COVID-19 patients could participate in massive recruitment of immune cells in the lung, contributing to lung injury and prolonged MV. However, massive endothelial adhesion (high expression of VCAM1) with less extensive diapedesis (lower level of sICAM1 in COVID-19 compared to non-COVID-19 patients) could also contribute to the massive sequestration of immune cells in the endothelial compartment, consequently contributing to a higher risk of thrombosis (36).

Accordingly, anti-VCAM1 humanized monoclonal antibody (36) could represent a promising therapeutic strategy, in order to dampen coagulopathy and improve outcomes in severe forms of COVID-19. However, since VCAM1 expression is induced by inflammatory mediators (e.g. TNF-α, IL-1β, IFN-α and γ) (32, 39), targeting the immune response is also another possible way to attenuate the deleterious effect of VCAM1-mediated endothelial and lung injury. Interestingly, we observed that sVCAM-1 was highly correlated with GM-CSF, the T_H_1 chemokine CXCL10 and IL-10, which our team and others recently identified as the peculiar “cytokine/chemokine signature” associated with poorer outcome in COVID-19 patients (13, 14, 16). A more robust T_H_1 immune response, and subsequent myeloid cell activation in COVID-19 could explain this peculiar cytokine signature, in which VCAM1 could be the consequence, but also a protagonist of the auto-inflammatory loop mediating endothelial and lung immune cell infiltration and tissue damage. Mitigating this immune response could also be a promising therapeutic avenue, since targeting upstream mediators would impact on both immune dysregulation and coagulopathy.

Our study may suffer from a lack of power given the large number of variables studied. However, it was an exploratory study in the context of a pandemic emergency and the multiplicity of tests was accounted for with the appropriate statistical techniques. In addition, we used several statistical methods to assess the association between COVID-19 status and biomarkers, for which data are scarce in the available literature. Second, we were unable to measure VCAM-1 expression in the tissues, even though no study to date dealing with COVID-19 was able to assess such data. This was a single center study with a small sample size and a short follow-up. A control group with non-severe COVID-19, and a follow-up measurement of clinical and biological data would have been more accurate to better assess the prognostic value of plasma biomarkers of coagulopathy in COVID-19. Since every patient did not receive systematically CT-pulmonary angiography and ultrasonography, venous thrombotic events may have been underestimated. Finally, we did not measure all the paths involved in coagulopathy (e.g. complement, or other markers of platelet activation), although we did observe lower levels of sP-selectin in COVID-19 patients, suggesting lower levels of platelet activation (4, 29). In addition, other biomarkers of endotheliopathy, such as angiopoietin 2, would have been worth evaluating in our study since it was found as a predictive biomarker of ICU admission in COVID-19 patients (40).

In conclusion, COVID-19 patients displayed a distinct coagulopathy signature with significantly higher concentrations of sVCAM1, but lower concentrations of some classical biomarkers of coagulation or platelet activation, compared to non-COVID-19 patients. This could result into a higher level of immune-mediated endotheliopathy. sVCAM1 was independently associated with the duration of MV. Further research is needed to investigate the role of VCAM-1 (or the immune response driving its expression), in the pathogenesis of severe COVID-19.

## Data Availability Statement

The original contributions presented in the study are included in the article/Supplementary Material, further inquiries can be directed to the corresponding author/s.

## Ethics Statement

The studies involving human participants were reviewed and approved by (Comité de Protection des Personnes SUD MEDITERRANEE V; 2017-A03404-49). Oral consent was obtained from the patient or their legal representatives.

## Author Contributions

MB, AB, CB, and LP: Concept and design. MB, J-PQ, MN, BB, and P-EC: Recruitment of patients. MB, EM, AB, CB, and LP: Acquisition, analysis, or interpretation of data. MB, EM, and AB: Drafting of the manuscript. MB, EM, AB, J-PQ, MN, BB, P-EC, CB, and LP: Critical revision. CB and LP: Supervision. All authors contributed to the article and approved the submitted version.

## LYMPHONIE STUDY GROUP CONTRIBUTORS

Pascal Andreu, François Aptel, Auguste Dargent, Marie Labruyère, Audrey Large, Sébastien Prin, (Department of Intensive Care, Dijon Bourgogne University Hospital, Dijon, France); Philippe Bonniaud, Guillaume Beltramo, Marjolaine Georges (Department of Pneumology, Dijon Bourgogne University Hospital, Dijon, France); Philip Bielefeld, Hervé Devilliers, Suzanne Mouries-Martin (Department of Internal Medicine and Systemic Diseases, Dijon Bourgogne University Hospital, Dijon, France); Bernard Bonnotte, Alexandre Guilhem (Department of Internal Medicine and Clinical Immunology, Dijon Bourgogne University Hospital, Dijon, France); Pascal Chavanet, Marielle Buisson (Infectious Diseases Department, Dijon Bourgogne University Hospital, Dijon, France); Alain Putot, Jeremy Barben (Geriatrics Internal Medicine Department, Dijon Bourgogne University Hospital, Dijon, France), Serge Monier (cytometry platform, Faculty of Medicine, Dijon, France).

## Conflict of Interest

The authors declare that the research was conducted in the absence of any commercial or financial relationships that could be construed as a potential conflict of interest.

## Publisher's Note

All claims expressed in this article are solely those of the authors and do not necessarily represent those of their affiliated organizations, or those of the publisher, the editors and the reviewers. Any product that may be evaluated in this article, or claim that may be made by its manufacturer, is not guaranteed or endorsed by the publisher.

## Abbreviations

ARDS: acute respiratory distress syndrome
ASAT: aspartate aminotransferase
COVID-19: coronavirus disease 2019
CXCL: C-X-C motif chemokine ligand
FDR: false discovery rate
GM-CSF: Granulocyte-macrophage colony-stimulating factor
ICU: intensive care unit
IQR: interquartile range
IL: interleukin
MV: mechanical ventilation
NT-ProBNP: N-Terminal Fragment of the Prohormone Brain-Type Natriuretic Peptide
PaO_2_:FiO_2_: arterial pressure of oxygen / oxygen inspiratory fraction
PDGF: platelet derived growth factor
PT: prothrombin time
RT-PCR: reverse transcriptase polymerase chain reaction
SAPS II: simplified acute physiology score II
SARS-CoV-2: severe acute respiratory syndrome coronavirus 2
SOFA: Sequential Organ Failure Assessment
vWF-A2: von Willebrand factor A2
uPA: u-plasminogen activator
TFPI: tissue factor pathway inhibitor
sTM: soluble thrombomodulin
sTREM-1: soluble triggering receptor expressed on myeloid cells 1
sICAM-1: soluble intercellular adhesion molecule 1
sVCAM-1: soluble vascular cell adhesion molecule 1
VEGF: vascular endothelial growth factor.
