# Supplementary material for: Specific Features of the Coagulopathy Signature in Severe COVID-19 Pneumonia
Source: Front Med (Lausanne). 2021 Aug 4;8:675191. doi: 10.3389/fmed.2021.675191 (PMC8371474; doi:10.3389/fmed.2021.675191)
Supplement: Supplementary file 1 [file Data_Sheet_1.docx]

**Specific Features of the Coagulopathy Signature in severe COVID-19 Pneumonia**

Mathieu Blot, MD, PhD, Emmanuel de Maistre, MD, Abderrahmane Bourredjem, MSc, Jean Pierre Quenot, MD, PhD, Maxime Nguyen, MD, Belaid Bouhemad, MD, PhD, Pierre-Emmanuel Charles, MD, PhD, Christine Binquet, MD, PhD, Lionel Piroth, MD, PhD, the LYMPHONIE study group.

**Supplementary files**

**Supplementary Table 1.** **Microbiological etiologies of severe pneumonia from 63 patients (Lymphonie study 2018-2020).**

|  | **Non Covid-19** | | **Covid-19** | | **Total N=63** | |
| --- | --- | --- | --- | --- | --- | --- |
|  | **N=36** | | **N=27** | |  | |
|  | **N** | **%** | **N** | **%** | **N** | **%** |
| **Microbiological pneumonia etiology** |  |  |  |  |  |  |
| Pure Bacterial | 10 | 28% | 0 |  | 10 | 16% |
| *Legionella pneumophila* | 3 | 8% | 0 |  | 3 | 5% |
| *Streptococcus pyogenes* | 2 | 6% | 0 |  | 2 | 3% |
| *Klebsiella pneumoniae* | 1 | 3% | 0 |  | 1 | 2% |
| *Mycoplasma pneumoniae* | 1 | 3% | 0 |  | 1 | 2% |
| *Streptococcus pneumoniae* | 1 | 3% | 0 |  | 1 | 2% |
| *Pseudomonas aeruginosa* | 1 | 3% | 0 |  | 1 | 2% |
| *Staphylococcus aureus* | 1 | 3% | 0 |  | 1 | 2% |
| Pure Viral | 10 | 28% | 26 | 96% | 36 | 57% |
| SARS-CoV-2 | 0 |  | 26 | 96% | 26 | 41% |
| Influenza A | 7 | 19% | 0 |  | 7 | 11% |
| Rhinovirus | 1 | 3% | 0 |  | 1 | 2% |
| Respiratory syncytial virus | 1 | 3% | 0 |  | 1 | 2% |
| Rhinovirus + seasonal coronavirus | 1 | 3% | 0 |  | 1 | 2% |
| Mixed | 3 | 8% | 1 | 4% | 4 | 6% |
| *Haemophilus parainfluenzae* + metapneumovirus | 1 | 3% | 0 |  | 1 | 2% |
| *Staphylococcus aureus* + influenza A | 1 | 3% | 0 |  | 1 | 2% |
| *Streptococcus pneumoniae* + influenza A | 1 | 3% | 0 |  | 1 | 2% |
| *Enterococcus faecium* + SARS-CoV-2 | 0 |  | 1 | 4% | 1 | 2% |
| Other non-documented | 13 | 36% | 0 |  | 13 | 21% |

**Supplementary Table 2.** **Biomarkers of coagulopathy (Lymphonie study 2018-2020)**

|  |  | **Study group** | | | |  |  |
| --- | --- | --- | --- | --- | --- | --- | --- |
|  |  | **non-COVID-19** | | **COVID-19** | | ***P-value*** | **FDR *Q-value*** |
|  |  | **N=36** | | **N=27** | |  |  |
| **Coagulation and endothelial activation** | |  |  |  |  |  |  |
| **D-dimers, ng/ml** | **mean ±SD** | 5,611 | ±1,932 | 4,542 | ±2,001 | **0.04** | 0.075 |
| **vWF-A2, pg/ml** | **mean ±SD** | 6,895 | ±3,561 | 4,727 | ±3,023 | **0.012** | **0.03** |
| **sTM, pg/ml** | **mean ±SD** | 10,924 | ±4,730 | 9,226 | ±3,373 | 0.104 | 0.15 |
| **sVCAM1, ng/ml** | **mean ±SD** | 3,700 | ±2,124 | 5,739 | ±3,293 | **0.009** | **0.028** |
| **sICAM1, ng/ml** | **mean ±SD** | 819.2 | ±376.4 | 579.9 | ±183.2 | **0.002** | **0.011** |
| **sTREM1, pg/ml** | **mean ±SD** | 910.5 | ±467.7 | 518.5 | ±190.2 | **<.0001** | **0.0005** |
| **TFPI, ng/ml** | **mean ±SD** | 43.5 | ±21.5 | 46.3 | ±20.1 | 0.596 | 0.677 |
| **uPA, pg/ml** | **mean ±SD** | 1,377 | ±491.2 | 1,335 | ±293.6 | 0.677 | 0.677 |
| **VEGF, pg/ml** | **mean ±SD** | 394.7 | ±288.3 | 231.3 | ±122.8 | **0.004** | **0.016** |
| **Platelet activation or growth factors** | |  |  |  |  |  |  |
| **CXCL4, ng/ml** | **mean ±SD** | 859.4 | ±691.1 | 1,473 | ±1,696 | 0.091 | 0.148 |
| **sP-selectin, ng/ml** | **mean ±SD** | 37.7 | ±9.9 | 32.1 | ±7.6 | **0.014** | **0.03** |
| **PDGF-AA, pg/ml** | **mean ±SD** | 2,453 | ±2,510 | 2,752 | ±2,863 | 0.667 | 0.677 |
| **PDGF-AB/BB, pg/ml** | **mean ±SD** | 652.3 | ±684.5 | 1,120 | ±1,627 | 0.169 | 0.219 |

COVID-19: coronavirus disease 2019; IQR: interquartile range; FDR: false discovery rate; SD: standard deviation.

**Supplementary Table 3. Principal component analysis (PCA) factor pattern correlation (LYMPHONIE study, 2018-2020).**

| **Factor Pattern** | **Factor1** | **Factor2** | **Factor3** |
| --- | --- | --- | --- |
| **Clinical features and outcomes** |  |  |  |
| Pneumonia Severity Index | **0.554** | . | . |
| SOFA score | **0.678** | . | . |
| ICU length of stay | 0.399 | . | **0.800** |
| Mechanical ventilation duration | 0.373 | . | **0.803** |
| Hospital length of stay | **0.433** | . | **0.704** |
| **Biological findings and immune cells** |  |  |  |
| PaO_2_:FiO_2_ | . | . | . |
| Lactate level | **0.657** | 0.306 | . |
| C-reactive protein | **0.560** | 0.376 | . |
| Procalcitonin | **0.748** | . | . |
| Serum Creatinine | **0.773** | . | . |
| NT-ProBNP | **0.673** | 0.376 | . |
| Leukocytes | . | **0.807** | . |
| Neutrophils | . | **0.804** | . |
| Monocytes | **-0.405** | **0.441** | . |
| Lymphocytes | **-0.501** | . | . |
| Platelets | **-0.438** | **0.561** | . |
| PT | **-0.409** | **-0.405** | 0.392 |
| **Coagulation and endothelial activation and growth factor** | | | |
| D-dimer | **0.462** | . | . |
| vWF-A2 | **0.580** | . | . |
| sTM | **0.764** | . | . |
| sVCAM1 | 0.320 | . | **0.467** |
| sICAM1 | **0.597** | 0.326 | . |
| sTREM1 | **0.772** | . | . |
| TFPI | **0.447** | . | . |
| uPA | **0.518** | -0.310 | . |
| VEGF | **0.638** | . | . |
| **Platelet activation or growth factors** |  |  |  |
| CXCL4 | . | **0.502** | 0.346 |
| sP-selectin | . | **0.490** | . |
| PDGF-AA | **.** | **0.762** | . |
| PDGF-AB/BB | . | **0.738** | 0.357 |

The table shows results of principal component analysis (PCA) including 30 variables (clinical characteristics and outcomes (n=5), biological findings (n=12), coagulation, endothelium or platelet biomarkers (n=13)). For clarity, we present only results for magnitude of loading of at least 0.3 and in bold, results above 0.4.

SOFA, Sequential Organ Failure Assessment; PaO_2_:FiO_2_, arterial pressure of oxygen / oxygen inspiratory fraction; ICU, intensive care unit; NT-proBNP, N-Terminal Fragment of the Prohormone Brain-Type Natriuretic Peptide.

**Supplementary Table 4. Spearman correlation between biomarkers of coagulopathy and severity (PSI, SOFA scores, PaO_2_:FiO_2_ ratio) or outcome (duration of mechanical ventilation) (Lymphonie study 2018-2020).**

|  | **PSI score** | | **SOFA score** | | **PaO_2_:FiO_2_ ratio** | | **Mechanical ventilation duration** | |
| --- | --- | --- | --- | --- | --- | --- | --- | --- |
|  | **r** | **P** | **r** | **P** | **r** | **P** | **r** | **P** |
| **D-dimer** | 0.15379 | 0.2327 | 0.20582 | 0.1085 | -0.03029 | 0.8152 | -0.06616 | 0.6094 |
| **vWF-A2** | **0.36654** | **0.0034** | 0.18810 | 0.1432 | 0.00441 | 0.9729 | 0.02342 | 0.8566 |
| **sTM** | **0.54691** | **<.0001** | **0.25958** | **0.0416** | -0.05382 | 0.6778 | 0.09192 | 0.4774 |
| **sVCAM1** | 0.19008 | 0.1389 | **0.32108** | **0.0109** | 0.04817 | 0.7100 | **0.50558** | **<.0001** |
| **sICAM1** | **0.28005** | **0.0275** | **0.26312** | **0.0388** | **-0.29052** | **0.0220** | -0.01363 | 0.9163 |
| **sTREM1** | **0.49538** | **<.0001** | 0.16808 | 0.1916 | -0.16350 | 0.2042 | -0.13004 | 0.3137 |
| **TFPI** | -0.01992 | 0.8779 | 0.18861 | 0.1421 | -0.02833 | 0.8270 | 0.14055 | 0.2759 |
| **uPA** | -0.02157 | 0.8678 | **0.36477** | **0.0036** | -0.09048 | 0.4843 | **0.39965** | **0.0013** |
| **VEGF** | **0.25357** | **0.0449** | 0.19044 | 0.1349 | -0.04287 | 0.7387 | -0.08457 | 0.5099 |
| **CXCL4** | 0.01421 | 0.9127 | **-0.25137** | **0.0488** | 0.10594 | 0.4125 | -0.13051 | 0.3120 |
| **sP-selectin** | 0.04413 | 0.7334 | 0.07836 | 0.5449 | -0.18743 | 0.1446 | -0.09961 | 0.4412 |
| **PDGF-AA** | 0.00374 | 0.9768 | **-0.25204** | **0.0463** | 0.11833 | 0.3557 | -0.14529 | 0.2559 |
| **PDGF-AB/BB** | 0.01123 | 0.9304 | -0.24624 | 0.0517 | 0.04964 | 0.6993 | -0.00952 | 0.9410 |

PSI: pneumonia severity index; SOFA: sequential organ failure assessment

**Supplementary Table 5. Multivariable linear regression factors associated with plasma sVCAM1 concentration among 63 patients with severe pneumonia (R²=24.6%) (LYMPHONIE study, 2018-2020).**

| **Variable** | **Mean diff** | **±SE** | **P** |
| --- | --- | --- | --- |
| **COVID-19** (Yes/No) | 2,247 | 695.4 | **0.002** |
| **Sex** (Male/Female) | 617.8 | 777.9 | 0.43 |
| **Age** (per additional year) | 41.1 | 29.6 | 0.170 |
| **SOFA score** (for each additional point) | 279.6 | 114.7 | **0.018** |
| **Charlson score** (for each additional point) | 23.9 | 222.6 | 0.91 |

COVID-19: coronavirus disease 2019; SOFA: Sequential Organ Failure Assessment; SE: standard error; sVCAM1: soluble vascular cell adhesion molecule 1.

**Supplementary Figure 1.** **Principal component analysis screen plot (retained dimension = 4)**

**Supplementary Figure 2.**
